# Retraction: In Vitro antioxidant and anticancer activity of young Zingiber officinale against human breast carcinoma cell lines

**DOI:** 10.1186/1472-6882-12-206

**Published:** 2012-11-02

**Authors:** Shahedur Rahman, Faizus Salehin, Asif Iqbal

**Affiliations:** 1 Department of Biotechnology and Genetic Engineering, Islamic University, Kushtia, 7003, Bangladesh; 2 Department of Biotechnology and Genetic Engineering, University of Development Alternative, Dhaka, Bangladesh

## 

The journal has retracted this article [1] because it contains large portions of text that were duplicated from articles previously published in The Journal of Medicinal Plants Research [2] and Molecules [3].

## Pre-publication history

The pre-publication history for this paper can be accessed here:

http://www.biomedcentral.com/1472-6882/12/206/prepub
